# Sars-Cov-2 Infection as Catecholamin Crisis in Pheocreomocitoma: A Case Report

**DOI:** 10.2174/0118715303348376250103113426

**Published:** 2025-02-10

**Authors:** Roberto Novizio, Andrea Corsello, Gaetano Emanuele Rizzo, Alfredo Pontecorvi, Pietro Locantore

**Affiliations:** 1 Unit of Endocrinology and Diabetology, Catholic University of The Sacred Heart, Policlinico Universitario “A. Gemelli” IRCCS, Rome, Italy;; 2 Unit of Endocrinology, Ospedale Fatebenefratelli Gemelli Isola, Rome, Italy;; 3 Unit of Endocrinology, Metabolism and Nutrition, Ospedale Paolo Borsellino, Marsala, Italy

**Keywords:** Pheochromocytoma, adrenal gland, covid-19, adrenal crisis, catecholamine release, alpha-blocker dosage

## Abstract

**Background:**

The primary presentation of SARS-CoV-2 infection is viral pneumonia, which may be complicated by acute respiratory distress syndrome, although several other manifestations can occur.. Endocrine implications have been described. Pheochromocytomas are rare tumors mainly originating in the adrenal medulla. Symptoms are primarily due to catecholamine overproduction and abrupt release. Catecholamine release is unregulated and could be continuous or paroxysmal. Some conditions (*i.e.*, stress, physical exercise, or specific foods) can trigger catecholamine release. Sars-CoV-2 infections have not been previously described as precipitators of adrenergic crises in pheochromocytoma patients. In this study, we report a case of adrenal crisis of a patient affected by pheochromocytoma in the context of Sars-CoV-2 infection.

**Case Presentation:**

A 63-year-old Caucasian male known for right adrenal pheochromocytoma waiting for surgical removal was admitted to the Emergency Department (ED) in March 2021 for a fainting episode and hypertensive crisis that he never experienced before.

The patient had a known medical history of type 2 mellitus diabetes and hypercholesterolemia treated by slow-release metformin 500 mg/day and atorvastatin 40 mg/day and was not vaccinated for Sars-CoV-2. Two months before, the patient was hospitalized in another hospital for myocardial infarction with non-obstructive coronary arteries, and a chest-abdomen TC scan showed a right adrenal lodge occupied by coarse formation. In the 24-h urine sample, metanephrines were >5000 µg/24 h and Normetanephrines >2500 µg/24 h. Scintigraphy with ^123^I-Metaiodobenzylguanidine (MIBG) showed accumulation in right adrenal gland formation, confirming the suspicion of pheochromocytoma. No further areas of pathological uptake were present. Fort that, the patient was started on alpha-blockers (doxazosin 2 mg twice/day). Two weeks later, the patient was also prescribed metoprolol 50 mg twice/day.

When admitted to the Emergency Department (ED), Blood Pressure (BP) was 210/108 mmHg with a heart rate of 105 bpm. A routine nasopharyngeal swab for Sars-CoV-2 was performed, resulting positive. After an extra dosage of 2 mg of doxazosin and 20 mg of nifedipine, symptoms addressed to catecholamine release disappeared. Being positive for Sars-CoV-19, the patient was transferred to the infectious diseases department. High mean BP was demonstrated at the control profile. Doxazosin was increased to 4 mg twice a day with a good effect on BP and tachycardia. After 10 days, the SARS-CoV-2 swab result was negative, and the patient was discharged with normal vital parameters and instructions to continue the increased dose of doxazosin. No other crisis was reported until surgery, which was performed without any complications after 1 month.

**Conclusion:**

Since the adrenal crisis is a life-threatening condition, we suggest close BP monitoring and therapeutic adherence in patients with pheochromocytoma waiting for surgery and living in areas characterized by outbreaks of COVID-19 infection. Moreover, we suggest considering an increase in alpha-blocker dosage to prevent the crisis.

## 1 INTRODUCTION

SARS-CoV-2, identified as COVID-19 by the World Health Organization (WHO), is a highly pathogenic β-coronavirus that has led to a global pandemic. In symptomatic individuals, the infection usually presents as viral pneumonia, which may progress to acute respiratory distress syndrome. Additionally, COVID-19 can cause non-respiratory complications, such as thrombosis, loss of smell (anosmia), myocarditis, and other issues [1-4]. Endocrinological manifestations in COVID-19 patients have been reported less frequently. The pathogenesis of COVID-19 involves SARS-CoV-2 entering the body through the respiratory system, utilizing the Angiotensin-Converting Enzyme 2 (ACE2) as a receptor to invade host pneumocytes. Beyond pneumocytes, the virus can also interact with ACE2 expressed in various other tissues, including endocrine organs such as the pancreas, thyroid, testes, ovaries, pituitary, and adrenal glands [5, 6].

Pheochromocytomas are rare tumors originating in the adrenal medulla. They may be sporadic or occur in the context of a hereditary syndrome. Symptoms are due to catecholamine overproduction and abrupt release or to mass effect. Diagnosis is confirmed by raised plasma or urine metanephrines or catecholamines and it is followed by imaging that is necessary for tumor localization and disease staging. Surgery, when feasible, is the first line and definitive treatment [7]. The catecholamine release is autonomous and could happen continuously with sustained hypertension or intermittently with episodic symptoms. SARS-CoV-2 infections have not been previously described as a trigger of catecholamine crisis in pheochromocytoma. Herein, we report, to the best of our knowledge, one of the very first cases reported of catecholamine crisis in a patient with pheochromocytoma during SARS-CoV-2 infection. This case report was prepared following the CARE guidelines [8].

## 2 CASE REPORT

**Fig. (1):**
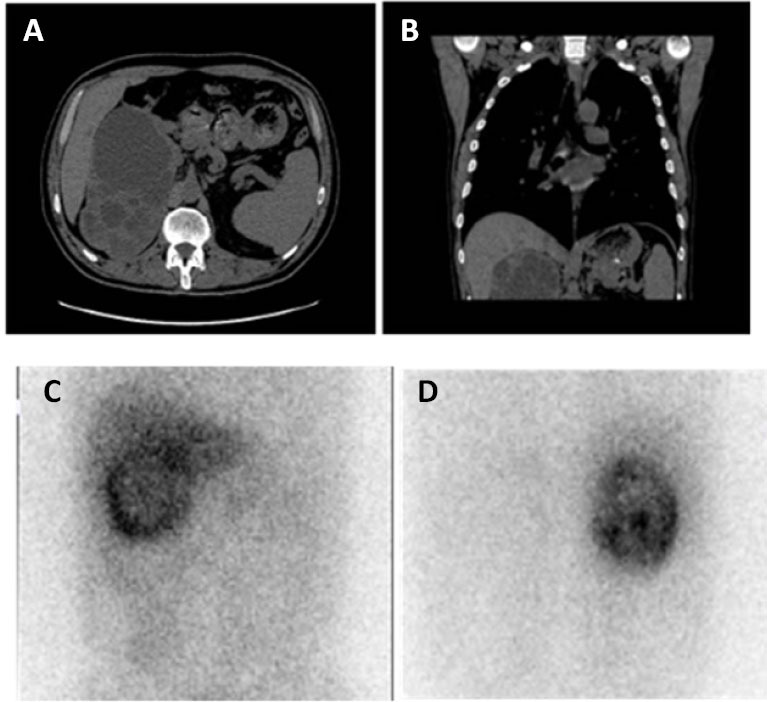
**(A-D)** Metaiodobenzylguanidine (MIBG), showing uptake in the right adrenal lesion, with no further areas of pathological uptake in the remaining areas of the body, compatible with the presence of a pheochromocytoma of the right adrenal site.

A 63-year-old Caucasian male, known for type 2 mellitus diabetes, microcytic anemia, and hypercholesterolemia, was hospitalized for Myocardial Infarction with Non-Obstructive Coronary Arteries (MINOCA) with a new onset of persistently high Blood Pressure (BP) and underwent complete cardiological work-up, including coronarography that showed no significant alteration of the coronary arteries tree in march 2021. A chest-abdomen CT was performed, showing the right adrenal lodge occupied by a coarse formation of 15x10x10 centimeters with inhomogeneous iso-hyperdense density, multi-chambered with internal septa and multiple cystic-like areas, some of which with thickened walls. The hormonal assessment included a 24-h urine sample, presenting Metanephrines >5000 µg/24h and Normetanephrine>2500 µg/24h. During hospitalization, the patient underwent medullary adrenal scintigraphy with 123 I- Metaiodobenzylguanidine (MIBG), showing an uptake in the right adrenal lesion, with no further areas of pathological uptake in the remaining areas of the body, compatible with the presence of a pheochromocytoma of the right adrenal site (Fig. **1A** and **B**). Therefore, the patient was started on doxazosin 2 milligrams [mg] per day with an indication for surgical removal after medical preparation. Two weeks later, due to inadequate BP control and increased heart rate, the patient was started on metoprolol 100 mg per day. Two months later, while waiting for surgery, the patient was admitted to the Emergency Department (ED) for a fainting episode and hypertensive crisis that he never experienced before. He reported a mild headache and weakness for 2 days previously. The patient was not vaccinated for SARS-CoV-2. At the admission to ED, the patient was taking cardio aspirin 100 mg per day, metformin 500 mg per day, pantoprazole 20 mg per day, doxazosin 2 mg per day, metoprolol 100 mg per day, and atorvastatin 40 mg per day. There was no relevant family history.

Upon his arrival, his blood pressure was 210/108 mmHg with a heart rate of 105 bpm. At admission, the patient had a routine nasopharyngeal swab for SARS-CoV-2. Blood tests showed electrolytes within normal range, normal hemocoagulation parameters, normal creatinine (0.98 mg/dl), and hyperglycemia (274 mg/dl). In ED, the patient underwent an electrocardiogram, showing sinus tachycardia with high T waves hyperkalemia-suggesting. Troponins evaluation showed increased values (68 ng/l, 267 ng/l, 73 ng/l). Echocardiography showed global kinetics within the limits, with no pericardial effusion. A few hours later, after an extra dosage of doxazosin 2 mg and nifedipine 20 mg, symptoms regressed, obtaining hemodynamic stability. Considering the recent coronarography that showed no significant alterations, the patient's symptoms were addressed to catecholamine release, so no further cardiological workup was conducted. Before his admittance to the ward, a nasopharyngeal swab resulted in positive for COVID-19, so he was transferred to the infectious diseases department. Neither fever nor COVID-19-related symptoms were objectified. A chest X-ray and, subsequently, a high-resolution CT scan were performed, showing in the apical segments of the inferior left lobe a small areola of increased density of the lung parenchyma with ground glass appearance associated with adjacent shaded micronodules, referred to as inflammatory/infectious alterations. Moreover, a coarse expansive formation with an inhomogeneous appearance, addressed to the known adrenal lesion of 15x10x10 centimeters, was documented in scans passing through the lung bases in the right adrenal lodge (Fig. **1C** and **D**). The blood pressure control profile showed high mean blood pressure and doxazosin was increased to 4 mg per day with beneficial effects on BP and tachycardia. After 10 days, a nasopharyngeal swab resulted negative for SARS-Cov-2, and the patient was discharged with normal vital parameters and an indication to continue endocrine and surgical evaluations in order to remove the pheochromocytoma. Upon discharge, the patient was instructed to continue antihypertensive therapy, including an increased dose of doxazosin, indicating a possible need for ongoing therapy despite clinical remission and COVID recovery, adding nifedipine to administer as needed in case of another hypertensive crisis. No other crisis was reported until surgery.

**Fig. (2):**
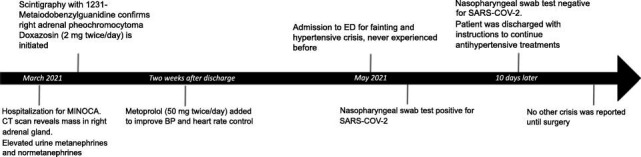
Timeline of patient’s key events.

The patient underwent a right laparoscopic adrenalectomy 1 month after the discharge. No surgical complication occurred (Fig. **2**). The patient was treated at Policlinico Universitario Agostino Gemelli – IRCCS in Rome.

## 3 DISCUSSION

Pheochromocytoma is a tumor arising from adreno-medullary chromaffin cells that commonly produce one or more catecholamines: epinephrine, norepinephrine, and dopamine. Pheochromocytomas can manifest as either paroxysmal or sustained hypertension, along with symptoms such as palpitations, tremors, sweating, headaches, and anxiety [9].

SARS-Cov-2 infection has several clinical presentations, ranging from asymptomatic patients to mild symptoms and acute severe respiratory stress. Herein, we reported a case in which COVID-19 probably triggered an adrenergic crisis in a patient with pheochromocytoma.

SARS-CoV-2 enters via the respiratory system using Angiotensin-Converting Enzyme 2 (ACE2) as an ingress receptor into host pneumocytes, but the virus can freely interact with receptors expressed in other tissues. The adrenal gland is one of the endocrine glands expressing ACE2, and it is involved in SARS-CoV-2 infection sequelae. One of the primary immune invasive strategies utilized by SARS-CoV-2 consists of knocking down the host’s cortisol stress response. A pathogenetic hypothesis proposed is the expression of certain amino acid sequences by the SARS-CoV-2 that are molecular mimics of the host Adrenocorticotropic Hormone (ACTH), stimulating antibodies production and destroying the circulating ACTH, then blunting the stress-induced cortisol rise. As a result, patients with severe COVID-19 may have a higher likelihood of developing corticosteroid insufficiency related to critical illness [3]. While many cases of adrenal insufficiency have been described in COVID-19 patients, hypersecretion of adrenal hormones is not typically considered.

If untreated, cardiovascular morbidity and mortality of pheochromocytoma are high. Exocytotic catecholamine release generally is episodic in nature. However, certain elements like stress, physical exercise, or even foods containing the molecule tyramine (*e.g.*, chocolate, cheese, or wine) can trigger the release of catecholamines. There are few reports of catecholamine crises probably triggered by infections and a possible role for the cytokines release during systemic infections has been postulated [10, 11]. In the last years, COVID-19 has been associated with several complications, but whether it can also trigger an adrenergic crisis in patients with pheochromocytoma has not been established. In theory, as any form of acute stress can be a trigger for catecholamine release, COVID-19 should be considered a possible cause. However, severe COVID-19 illness hinders the direct study of catecholamines in these patients, especially those on multiple medications, those on adrenaline or noradrenaline infusions, or both [12]. Also, glucocorticoids are often employed in patients with COVID-19, and the interactions with catecholamines are multiple. Indeed, while some reports suggest that exogenous corticosteroids may precipitate an adrenergic crisis, others indicate that corticosteroids can improve the patient's general condition, reduce inflammation, and decrease cytokine release, potentially mitigating the effects of catecholamine excess and theoretically lowering the risk of triggering catecholamine release [13]. Interestingly, to the best of our knowledge, only a few cases of catecholamine crisis during COVID-19 have been reported, and these were related to adrenal hemorrhage in a patient with incidental pheochromocytoma [14-16].

In our patient, the diagnosis of pheochromocytoma had already been established, and the patient was already on alpha-blockers. Since the disease was perfectly controlled by antihypertensive therapy, the patient did not refer to any changes in his lifestyle or drug assumption during the last two months. This could be the first case of COVID-19 infection as the precipitating cause of the adrenergic crisis in a pheochromocytoma-affected patient.

Finally, while this case report focuses on a male patient, it is valuable to consider whether the same therapeutic regimen might be similarly effective in female patients with pheochromocytoma experiencing a COVID-19-triggered catecholamine crisis. Research has shown some sex differences in pheochromocytoma presentations, with female patients occasionally exhibiting more pronounced symptoms related to catecholamine release. However, current literature does not indicate substantial differences in the response to alpha-blockers and beta-blockers between men and women with this condition [17, 18].

## CONCLUSION

Adrenergic crisis is a life-threatening condition. Patients with a history of pheochromocytoma surgery should monitor their blood pressure regularly at home and can be reassured that their risk of COVID-19 is not increased. If admitted with COVID-19, no special requirements should be necessary, but plasma and urinary metanephrines may be grossly elevated during severe disease, so they should not necessarily be indicative of recurrent tumors.

There are currently no specific drug-treatment protocols available for patients with COVID-19 and pheochromocytomas. We suggest close blood pressure monitoring and therapeutic adherence in patients with pheochromocytoma waiting for surgery living in areas characterized by outbreaks of COVID-19 infection. Moreover, we suggest considering an increase in alpha-blocker pharmacological dosage in order to prevent the crisis.

## LIST OF ABBREVIATIONS

WHO: World Health Organization
ACE2: Angiotensin-Converting Enzyme 2
MINOCA: Myocardial Infarction with Non-Obstructive Coronary Arteries
BP: Blood Pressure
ED: Emergency Department
ACTH: Adrenocorticotropic Hormone

## STANDARDS OF REPORTING

CARE guidelines were followed.
